# Direct cost of ultrasound-guided outpatient medical procedures for patients with pain

**DOI:** 10.1590/0034-7167-2025-0317

**Published:** 2026-06-22

**Authors:** Otávio Paino Paim, Roberto Del Valhe Abi Rached, Antônio Fernandes Costa Lima

**Affiliations:** I Universidade Santo Amaro. São Paulo, São Paulo, Brazil; II Universidade de São Paulo. São Paulo, São Paulo, Brazil

**Keywords:** Ambulatory Care, Acute Pain, Chronic Pain, Ultrasonic Therapy, Direct Service Costs., Atención Ambulatoria, Dolor Agudo, Dolor Crónico, Terapia por Ultrasonido, Costos Directos de Servicios.

## Abstract

**Objectives:**

to measure the mean direct costs related to labor and supplies required for performing ultrasound-guided outpatient medical procedures for patients with pain.

**Methods:**

quantitative, exploratory-descriptive, prospective micro-costing research, of the single case study type, conducted in a pain clinic from October 2024 to January 2025.

**Results:**

the human resources and supplies (materials, medications, and solutions) consumed in 82 procedures were documented, resulting in the following mean total direct costs: joint aspiration - US$30.40 (SD=26.86); nerve block - US$14.47 (SD=2.74); cervical and lumbar spine infiltrations - US$24.92 (SD=23.62); trigger point inactivation - US$12.90 (SD=2.81); and interfascial plane block - US$15.88 (SD=11.73). The greatest impact on costs resulted from medication/solution and material consumption.

**Conclusions:**

measuring the mean direct costs of the aforementioned procedures contributes to assessing the cost-benefit ratio associated with them, providing financial support for clinical-administrative decisions aimed at the appropriate pain treatment/management.

## RESUMEN

**Objetivos:**

medir los costos directos promedio relacionados con la mano de obra y los insumos necesarios para realizar procedimientos médicos ambulatorios guiados por ultrasonido en pacientes con dolor.

**Métodos:**

estudio cuantitativo, exploratorio-descriptivo, prospectivo de microcosteo, de tipo caso único, realizado en una clínica del dolor desde octubre de 2024 hasta enero de 2025.

**Resultados:**

se documentaron los recursos humanos y los suministros (materiales, medicamentos y soluciones) consumidos en 82 procedimientos, obteniéndose los siguientes costos directos totales promedio: punción articular - US$30,40 (DE=26,86); bloqueo nervioso - US$14,47 (DE=2,74); infiltraciones de la columna cervical y lumbar - US$24,92 (DE=23,62); inactivación de puntos gatillo - US$12,90 (DE=2,81); y bloqueo interfacial - US$15,88 (DE=11,73). El mayor impacto en los costos se debió al consumo de medicamentos/soluciones y materiales.

**Conclusiones:**

la medición de los costos directos promedio de los procedimientos mencionados contribuye a evaluar la relación costo-beneficio asociada, brindando respaldo financiero para las decisiones clínico-administrativas dirigidas al tratamiento/manejo adecuado del dolor.

## INTRODUCTION

The International Association for the Study of Pain (IASP) describes that pain is a negative sensory and emotional experience that is related to tissue damage, whether actual or potential^(1)^.

A systematic review, which analyzed 35 studies investigating the prevalence of chronic pain in Brazil, showed a variation between 23.02% and 76.17%, with a national mean of 45.59%, affecting more women than men. A higher prevalence was observed in the Central-West region (56.25%), but the Southeast region had the most studies and the largest population analyzed (42.2%). Regarding the IASP classifications of pain mechanisms, possibly nociceptive pain had a prevalence of 36.70%, neuropathic pain of 14.5%, and nociplastic pain of 12.5%^(2)^.

Chronic pain can be aggravated by psychiatric illnesses, as the sensitivity to pain in patients with illnesses such as depression is greater than in an individual free from adverse mental conditions, with continuous stress being an influence on pain^(3)^. Excessive secretion of stress hormones such as cortisol and catecholamines is a significant factor in low back pain and fibromyalgia^(4)^.

Acute pain is characterized by a sudden onset, usually associated with a specific event or injury^(5)^. It works as a protective response, activating peripheral nociceptors and transmitting pain signals to the central nervous system^(6)^. While it usually resolves within a short period of time as the body heals, acute pain can be severe and hinder recovery. In some cases, if not managed effectively, it can become chronic pain, adding challenges to patient care and recovery, especially in post-surgical cases^(7)^.

The complexity of pain perception and its multidimensional nature show the importance of a comprehensive approach to its management^(8)^, whether pharmacological or through minimally invasive procedures, which, depending on the pain type and site, are considered more effective^(9)^.

Among invasive treatments, peripheral nerve stimulation, which has the potential to reach the affected area, causes pain reduction by stimulating the nerve in the injured tissue^(10,11)^. Other therapeutic techniques are also effective, such as interfascial plane block, injection in regional sites, intra-articular injection of drugs, and the use of anti-inflammatory drugs with or without the addition of opioid anesthetics^(11)^.

Fluoroscopy, which uses real-time X-rays, has become an important tool in interventional pain procedures such as nerve blocks and epidural injections, allowing for precise and immediate visualization, much like ultrasound. Comparing the use of fluoroscopy and ultrasound in the postoperative period, fluoroscopy was shown to be superior in the Oswestry Disability Index, which measures functional restrictions caused by pain in people with low back pain. However, this superiority has no significant clinical implications, with both methods being safe for vasovagal reactions, transient headache, and facial flushing^(12)^.

Interventional radiology plays a crucial role in painful bone metastasis treatment, using MRI-guided techniques, preserving healthy tissue, with a faster recovery time and fewer post-operative complications^(13)^. A systematic review demonstrated that recent studies demonstrate the clinical effectiveness of ultrasound-guided procedures in chronic pain treatment, making pain management services more cost-effective^(14)^.

Post-surgical pain management has been more precisely tailored to individual patient needs, through a combination of different therapeutic methods aimed at reducing side effects and increasing routine patient contact. This promotes more effective and safer treatment, with constant monitoring and adjustments as needed^(15)^.

The various interventions to treat pain require the consumption of specialized labor (LAB) and supplies (medications, solutions, and materials) whose costs need to be properly calculated and managed in order to maintain the financial sustainability of the relationship established between healthcare providers and payers, whether they are from the Supplementary Health System (In Portuguese, *Sistema de Saúde Suplementar* - SSS) (insurance companies, health plan operators (HPOs), and/or private patients) or the Brazilian Health System (In Portuguese, *Sistema Único de Saúde* - SUS).

Given the situation of scarce resources and growing demands for healthcare services, process and/or procedure costing is fundamental for the effectiveness, efficiency, and continuous improvement of the quality of health systems^(16)^. It assists in controlling operational costs by identifying areas where there are excessive expenses or inefficiencies, and allows for the implementation of corrective measures aimed at preventing waste, losses, and obsolescence that result in unnecessary costs. It contributes to the decision-making process regarding investments in new technologies, more effective and economical treatment methods and care models, the development of new services, and the improvement of processes^(17,18)^. From this perspective, the present study was carried out.

## OBJECTIVES

To measure the mean direct costs (MDCs) related to LAB and supplies required for performing ultrasound-guided outpatient medical procedures for patients with pain.

## METHODS

### Ethical aspects

This study was approved by the Research Ethics Committee of the School of Nursing at the *Universidade de São Paulo*.

### Study design, location and data collection period

This is exploratory-descriptive, prospective micro-costing research^(16,19)^, of the single case study type^(20)^, conducted according to the Equator Network CARE instrument guidelines, in a private pain clinic located in the Bela Vista neighborhood, in the city of São Paulo.

A pain clinic was selected for convenience, given its adequate infrastructure for outpatient pain care and the fact that one of the authors works there as a medical intern. It features a procedure room equipped with advanced imaging technology to guide interventional procedures and an office for appointments and medical assessments.

The center assists approximately 300 patients per month, both private and HPOs. Approximately 80% of patients receive treatment with ultrasound-guided procedures. The most prevalent pathologies are cervical and low back pain, knee osteoarthritis, myofascial pain, neuropathies, tendonitis, and bursitis. The center has three pain specialists and, when necessary, recommends other rehabilitation professionals (physiotherapists, occupational therapists, psychologists, rheumatologists, orthopedists, and psychiatrists) to patients and their family members/legal guardians for support with the required treatment.

It should be noted that only one of the physicians performs ultrasound-guided outpatient procedures, assisted by a medical intern. Individualized treatment plans are implemented, tailored to each patient’s specific needs, integrating different therapeutic modalities for holistic pain management. Continuous monitoring is also provided, with reassessment of treatment plans to ensure their effectiveness.

At the pain clinic, the supply acquisition process follows a standardized flow to ensure the continuous availability of necessary materials and cost optimization. The medical director prepares a consumption forecast, taking into account the standardization of quantities used in each procedure and historical demand, including during peak periods.

Each week, the intern conducts a physical inventory of the remaining supplies and forwards the survey to the secretary, who formalizes the purchase request through the e-commerce platforms of medical supply distributors and websites of specific manufacturers for supplies.

Before placing an order, the medical director reviews the request to validate the quantities and approve the purchase. This method ensures strict inventory control, minimizes waste, and enables more advantageous negotiations with suppliers, contributing to the clinic’s financial sustainability and maintaining quality of care.

During the data collection period, the number of ultrasound-guided procedures ranged from 125 to 189, with a mean of 151. Data collection took place from October 2024 to January 2025. The dates and times for conducting non-participant observations were previously agreed upon with a pain specialist, respecting ethical precepts regarding the privacy of patients undergoing treatment and maintaining the anonymity of all those involved.

### Sample, inclusion and exclusion criteria

The case series corresponded to opportunities for direct, non-participant observation of the time spent by physicians, assisted by medical interns, and the consumption of supplies required to perform the following ultrasound-guided outpatient medical procedures for patients with pain: joint puncture; nerve blocks; infiltrations in the cervical and lumbar spine; trigger point inactivation; and interfascial plane blocks.

The observation sample size, to support direct costing, as previously advised by a statistician, was determined by non-probability convenience sampling, considering that the procedures would be performed by the same physician assisted by the same intern. Therefore, there would be no significant variations in the time spent by the performers (direct labor (DLAB)) or the consumption of medications, solutions, and materials. All patients treated during the data collection period were included in the study.

### Theoretical-methodological path

The single case study was adopted to support the empirical investigation of a given phenomenon, following a set of procedures. It is based on the logic of planning, which incorporates specific approaches related to data collection and analysis techniques from multiple sources of evidence in order to understand contemporary phenomena, based on their real-world context. It allows us to grasp the totality of a situation, describe, understand, and interpret the complexity of a specific case, delving deeper into a delimited object^(20)^.

It was based on the microcosting methodology, which uses detailed data on resource utilization and unit costs to generate accurate estimates of economic costs^(16,19)^. It consisted of measuring direct costs related to the consumption of human resources (DLAB), materials, medications, and solutions for performing ultrasound-guided outpatient procedures.

Direct costs are defined as those that can be clearly quantified and identified. They refer to monetary expenditures incurred in the production of a product/service that can be identified with the product or department^(21)^. In hospital organizations, direct costs are made up of the DLAB, supplies and equipment used in the care process^(22)^.

Performing a given procedure requires the consumption of varying amounts of resources. The MDC of a procedure is calculated, from the sum of the MDC of the materials, of solutions/medications and LAB^(23)^.

To obtain the total MDC of the procedures under study, the mean quantity of materials, the mean unit price of each material, the mean amount of solutions/medications, the mean unit price of each solution/medication, the mean time dedicated to each professional category, and the mean unit wage cost of the LAB of each professional category^(20)^ were defined according to the equation as follows^(23)^:.

The values obtained in *reais* (R$ (Brazilian currency)) were converted to US dollars (US$) considering the mean conversion rate of R$ 5.96/US$ 1.00 for the period from October 2024 to January 2025.

The pain clinic’s medical director provided information regarding the types/presentations of materials, and solutions/medications and costs of the most recent acquisitions, as well as the salaries and monthly working hours of physicians (R$20,000.00/150 hours/month) and intern (R$500.00/50 hours/month) involved in the procedures. Then, they obtained the values per hour (R$133.33 and R$10.00) and per minute (R$2.22 and R$0.17).

After being organized in a spreadsheet, the data was transported to the bottom-up direct microcosting simulator^(24)^, from the Research Group on the Economic Dimension of Nursing Management, and treated using descriptive and inferential statistics.

## RESULTS

Non-participant observations were conducted of five outpatient medical procedures, guided by ultrasound, aimed at treating 82 patients with pain. The ages of these patients ranged from 33 to 85 years, with a mean of 58.04 (SD = 15.85) years. Furthermore, 58.03% were female and 41.97% were male.

The procedures lasted between five and 13 minutes, with a mean of 9.62 (SD=1.69) minutes. There was a wide variety of medical diagnoses, and the most frequent, according to the International Classification of Diseases-10, corresponded to: 12 patients with G64.1 (other disorders of the peripheral nervous system); 12 patients with M53.1 (cervicobrachial syndrome); six patients with M707 (other bursitis of hip); and five patients with M54.5 (low back pain).

Table 1 indicates that the total MDC of the joint aspiration procedure was US$30.40 (SD=26.86), ranging from US$5.47 to US$77.45. The MDC including DLAB totaled US$4.18, with the MDC of medications, solutions, and materials (US$26.22) accounting for 86.47% of the total MDC.

**Table 1:** Cost distribution, in US dollars (US$^*^), of direct labor of physicians and medical interns, and of the medications, solutions, and materials consumed in 17 ultrasound-guided joint puncture procedures for patients with pain, São Paulo, São Paulo, Brazil, 2025

Variables	Mean US$^*^	Standard deviation US$^*^	Minimum US$^*^	Maximum US$^*^	Median US$^*^
Direct labor cost with physicians	3.82	0.55	2.61	4.85	3.73
Direct labor cost with interns	0.36	0.05	0.24	0.45	0.35
Mean direct cost with medications, solutions, and materials	26.22	26.81	2.61	73.37	10.13
**Total mean direct cost**	**30.40**	**26.86**	**5.47**	**77.45**	**14.70**

* *Central Bank of Brazil: mean exchange rate of R$5.96/US$1.00 for the period from October 2024 to January 2025.*

In the 17 joint aspirations observed, the prevailing costs were associated with the consumption of injectable Osteonil Plus^®^ 40 mg (unit price of US$49.33), Synollis^®^ 40/80 mg/ml (US$65.44), Triancil^®^ 20 mg/mL (unit price of US$4.66), and levobupivacaine hydrochloride in enantiomeric excess without vasoconstrictor 0.50% 5 mg/mL (unit price of US$4.53).

Table 2 shows that the total MDC of nerve blocks was US$14.47 (SD=2.74), with a minimum of US$11.58 and a maximum of US$21.03. The MDC with the DLAB was US$3.86, highlighting the MDC of medications, solutions, and materials (US$10.62), which accounted for 73.73% of the total MDC.

**Table 2:** Cost distribution, in US dollars (US$^*^), of direct labor of physicians and medical interns, and of the medications, solutions, and materials consumed in 16 ultrasound-guided nerve block procedures for patients with pain, São Paulo, São Paulo, Brazil, 2025

Variables	Mean US$^*^	Standard deviation US$^*^	Minimum US$^*^	Maximum US$^*^	Median US$^*^
Direct labor cost with physicians	3.50	0.53	2.61	4.47	3.54
Direct labor cost with interns	0.36	0.05	0.24	0.45	0.35
Mean direct cost with medications, solutions, and materials	10.62	2.60	7.52	16.99	9.62
**Total mean direct cost**	**14.47**	**2.74**	**11.58**	**21.03**	**13.57**

* *Central Bank of Brazil: mean exchange rate of R$5.96/US$1.00 for the period from October 2024 to January 2025.*

In the 16 nerve blocks observed, the predominant costs were associated with levobupivacaine hydrochloride in excess of enantiomeric purity without vasoconstrictor 0.50% 5 mg/mL - 20 mL (unit cost of US$4.53), lidocaine hydrochloride without vasoconstrictor 2.0% 20 mg/mL (unit cost of US$1.69), and 50% glucose - 10 ml (unit cost of US$0.80). Among the materials, the highest costs resulted from the consumption of 25G x 60 Nanoneedle Japanese Steel^®^ needles (unit cost of US$2.25).

Table 3 shows that the total MDC amounted to US$24.92 (SD=23.62), ranging from US$6.78 to US$85.59. The MDC including DLAB totaled US$4.18, with the MDC of medications, solutions, and materials (US$20.74 - 83.46%) being the most representative component of the total MDC.

**Table 3:** Cost distribution, in US dollars (US$^*^), of direct labor of physicians and medical interns, and of the medications, solutions, and materials consumed in 16 ultrasound-guided cervical and lumbar spine injection procedures for patients with pain, São Paulo, São Paulo, Brazil, 2025

Variables	Mean US$^*^	Standard deviation US$^*^	Minimum US$^*^	Maximum US$^*^	Median US$^*^
Direct labor cost with physicians	3.82	0.60	2.98	4.85	3.73
Direct labor cost with interns	0.36	0.06	0.28	0.45	0.35
Mean direct cost with medications, solutions, and materials	20.74	23.64	2.70	81.92	10.87
**Total mean direct cost**	**24.92**	**23.62**	**6.78**	**85.59**	**13.56**

* *Central Bank of Brazil: mean exchange rate of R$5.96/US$1.00 for the period from October 2024 to January 2025.*

In the 16 cervical and lumbar spine infiltrations observed, costs related to the consumption of Triancil^®^ 20 mg/mL (unit price of US$4.66), levobupivacaine hydrochloride in enantiomeric excess without vasoconstrictor 0.50% 5 mg/mL (unit price of US$4.53), and methylprednisolone acetate 40 mg/mL - 2 mL (unit price of US$4.51) stood out.

According to Table 4, the MDC amounted to US$12.90 (SD = US$2.81), with a minimum of US$7.06 and a maximum of US$18.03. The MDC including DLAB totaled US$3.59, with a predominance of the MDC of medications, solutions and materials (US$9.31) accounting for 72.48% of the total MDC composition.

**Table 4:** Cost distribution, in dollars (US$^*^), of direct labor of physicians and medical interns, and of the medications, solutions, and materials consumed in 16 ultrasound-guided trigger point inactivation procedures for patients with pain, São Paulo, São Paulo, Brazil, 2025

Variables	Mean US$^*^	Standard deviation US$^*^	Minimum US$^*^	Maximum US$^*^	Median US$^*^
Direct labor cost with physicians	3.28	0.80	1.86	4.47	3.54
Direct labor cost with interns	0.31	0.07	0.17	0.42	0.33
Mean direct cost with medications, solutions, and materials	9.31	2.90	2.98	13.95	8.83
**Total mean direct cost**	**12.90**	**2.81**	**7.06**	**18.03**	**12.60**

* *Central Bank of Brazil: mean exchange rate of R$5.96/US$1.00 for the period from October 2024 to January 2025.*

In the 16 trigger point inactivations observed, the predominant costs were associated with t levobupivacaine hydrochloride in enantiomeric excess without vasoconstrictor 0.50% 5 mg/mL (unit cost of US$4.53) and lidocaine hydrochloride without vasoconstrictor 2.0% 20 mg/mL (unit cost of US$1.69). Regarding materials, the highest costs were associated with the consumption of 25G x 60 Nanoneedle Japanese Steel^®^ (unit cost of US$2.25) and Raquidiana Spinal Bd^®^ needles - 25G x 3 1/2 (unit cost of US$1.64).

Finally, Table 5 shows that the total MDC for interfascial plane block was US$15.88 (SD = US$11.73), ranging from US$6.02 to US$59.54. The MDC with DLAB corresponded to US$3.86, and the MDC of medications, solutions, and materials (US$12.02) accounted for 76% of the total MDC composition.

**Table 5:** Cost distribution, in US dollars (US$^*^), of direct labor of physicians and medical interns, and of the medications, solutions, and materials consumed in 17 ultrasound-guided interfascial plane block procedures for patients with pain, São Paulo, São Paulo, Brazil, 2025

Variables	Mean US$^*^	Standard deviation US$^*^	Minimum US$^*^	Maximum US$^*^	Median US$^*^
Direct labor cost with physicians	3.53	0.53	2.61	4.47	3.73
Direct labor cost with interns	0.33	0.05	0.24	0.42	0.35
Mean direct cost with medications, solutions, and materials	12.02	11.54	2.35	55.05	9.84
**Total mean direct cost**	**15.88**	**11.73**	**6.02**	**59.54**	**13.24**

* *Central Bank of Brazil: mean exchange rate of R$5.96/US$1.00 for the period from October 2024 to January 2025.*

In the 17 interfascial plane blocks observed, the predominant costs were associated with the medications levobupivacaine hydrochloride in enantiomeric excess without vasoconstrictor 0.50% 5 mg/mL - 20 mL (unit value of US$4.53), injectable Osteonil Plus^®^ 40 mg (unit value of US$49.33), lidocaine hydrochloride without vasoconstrictor 2.0% 20 mg/mL (unit value of US$1.69), and with the material 25G x 60 Nanoneedle Japanese Steel^®^ needles (unit value of US$2.25).

## DISCUSSION

Conducting this unique case study allowed for the analysis of ultrasound-guided outpatient medical procedures performed on patients treated at a pain clinic. As anticipated in this approach^(20)^, relevant information was obtained, considering the specificity of its real-world context, to guide clinical decision-making, assess the need for developing new care strategies, and contribute to the improvement of professional practice.

The patients included in the study had a mean age of 58.04 (SD=15.85) years, with 58.03% being female, and presented with varied clinical diagnoses, predominantly musculoskeletal and peripheral neurological disorders. The most frequent classifications were G64.1 (other disorders of the peripheral nervous system), M53.1 (cervicobrachial syndrome), M70.7 (other bursitis of the hip), and M54.5 (low back pain). These diagnoses correspond to classic cases of musculoskeletal and neuropathic pain, frequently observed in rehabilitation settings.

The profile mentioned above is consistent with the results of a systematic review conducted in Brazil, which showed a higher prevalence of chronic pain among women, especially those aged between 45 and 66 years^(2)^. Another systematic review and meta-analysis examined the prevalence of chronic pain in the general adult population, finding it to be between 23.02% and 41.4%, associated with female sex and advanced age (29.3% to 76.2%). It concluded that chronic pain is highly prevalent, is associated with significant distress and disability, and has been poorly managed^(25)^.

The mean age of the sample analyzed reflects a stage of life in which musculoskeletal and peripheral neurological degenerative changes intensify, often associated with functional limitations. These findings highlight the need for specific therapeutic approaches aimed at pain management in this age group^(26)^. The study emphasizes that conditions such as tendinopathies, bursitis, and low back pain are highly prevalent and tend to respond well to local therapies^(11)^. Women with fibromyalgia, even those with higher levels of education, face significant functional and emotional impacts resulting from chronic pain^(27)^, reinforcing the relevance of specific therapies, such as the nerve blocks and infiltrations funded in this study.

In the calculation of total MDCs, there was no significant variation in the MDC with DLAB between the pain specialist physician and the medical student intern, which ranged from R$3.59 to US$4.18, with a mean of US$3.93. Despite the essential need for specialized DLAB from the performers, this MDC corresponded to the lowest representation in the overall composition of the total MDC of the procedures under study, corroborating the results of direct costing studies that demonstrated the prevalence of costs associated with materials and/or medications/solutions^(17,18)^.

The costs of joint aspiration (US$30.40) and trigger point inactivation (US$12.90) stood out, showing the highest and lowest total MDCs, respectively. This disparity is justified by the complexity of the procedure and the need for specific materials, which directly impact the cost of outpatient interventions^(28)^. In the case of joint aspirations, the highest cost was related to the use of specific medications such as Osteonil Plus^®^. The dependence on high-cost drugs reinforces the need for public policies that favor the acquisition of these supplies at affordable prices. With this intention, efficient management of hospital purchases can significantly reduce the unit cost of high-value materials^(29)^.

Concerning nerve blocks, although they also require medications such as levobupivacaine and lidocaine, the MDC was relatively low (US$14.47), indicating a good cost-benefit ratio for this procedure, also considering its therapeutic efficacy reported in the literature. An integrative review shows that ultrasound-guided procedures provide significant pain relief, reduce the need for opioids, and improve the functional recovery of patients^(14)^.

Infiltrations in the spine showed a high MDC (US$24.92), with variation among the observed cases (SD=US$23.62), which points to heterogeneity in the therapeutic choice or in the individual response to treatment. This aligns with what was observed in a study that highlights the importance of therapeutic personalization in interventional pain treatments, even though this represents facing challenges in cost control^(30)^.

Trigger point inactivation and interfascial plane blocks stood out for having more uniform and relatively lower costs. However, it is reiterated that, in the five procedures studied, the MDCs containing medications, solutions, and materials predominated in the composition of the total MDCs.

Therefore, the importance of adequate management of material resources is corroborated to identify, analyze, and reduce waste and losses resulting in costs that, if avoided, could be redirected to the benefit of other patients, contributing to the efficiency of processes and financial sustainability of healthcare organizations^(31)^. In this regard, rationalization strategies in the acquisition and prescription of medications are essential^(32)^.

It is known that more complex procedures, which require specialized DLAB and a greater quantity of materials, medications, and solutions, result in higher costs. Therefore, it is necessary to consider the complexity of the procedures and the specific resources in the planning and management of outpatient interventions, aiming at their optimization and contribution to the financial sustainability of healthcare services. Thus, careful planning of the required resources logistics, combined with their rational allocation and use, can favor increased access to these therapies in the outpatient setting. This perspective is consistent with the principles of health economics, which aim at optimizing resources based on clinical effectiveness^(28)^.

A systematic review showed that recent studies have highlighted the clinical effectiveness of ultrasound-guided procedures in chronic pain treatment. The literature indicates that pain management services can be cost-effective for the treatment of chronic low back pain, although there is variability in the evidence due to the quality of published studies and the diversity of interventions, comparators, and outcomes^(33)^.

The cost of the drugs used, especially the high-cost ones (Osteonil Plus^®^ and Synollis^®^), significantly influenced the MDCs of joint aspirations in this study. It is noteworthy that cost-sharing schemes, value-based pricing, reference pricing, reimbursement mechanisms, and the substitution of original medications with generics and biosimilars have been strategies to manage pharmaceutical expenditures, ensuring fair access to essential medications^(34)^.

In the Brazilian SSS, after assessing 79,689 patients with chronic pain over four years, it was found that 96% of patients underwent at least one examination, and 86% had at least one emergency consultation during the period. The total estimated cost for the SSS amounted to more than 3 billion reais. Experts pointed out that the fragmentation of the health system and the lack of patient-centered approaches contribute to the ineffective pain management, resulting in high resource utilization. They emphasized the importance of integrating clinical and direct cost data for more efficient resource management and increased access to interventional therapies^(32)^.

A thematic literature review demonstrated that the managerial implications of pricing healthcare services, especially in hospitals, have still received insufficient academic attention. By analyzing the understanding of the nature of hospital service pricing, the differentiation of pricing objectives, strategies, and practices, and the presentation of factors that impact hospital service pricing, it was indicated that hospital pricing is a complex and unclear issue. It has been influenced by costs, demand and supply factors, market structure, price regulation, and third-party reimbursements^(35)^.

Finally, it is confirmed that the microcosting method, often considered the “gold standard” due to its ability to provide detailed and accurate cost data, allows for a thorough analysis of each individual cost component of a service or treatment, encompassing medications, materials, equipment, and LAB, resulting in more reliable information and enabling a highly accurate assessment of the process or procedure. This precision is crucial for the economic assessment of health technologies, allowing the identification of which treatments or interventions are most cost-effective^(16)^.

### Study limitations

The single case study was a limitation of this study. For future research and the verticalization of the knowledge developed, multiple case studies are recommended, which will allow for the coverage of multiple dimensions and perspectives, whose comparison will strengthen the robustness of results.

### Contributions to nursing, health, or public policies

The results obtained contribute to proposing a rational basis to assist healthcare service administrators in justifying the pricing process for outpatient medical procedures guided by ultrasound, intended for patients with pain. From this perspective, it facilitates negotiation with payers, whether they are from the SSS, SUS, or private patients.

The methodology adopted in this study can be replicated in other healthcare services, regardless of their legal nature, as well as for procedures performed by other professional health categories, providing financial information that supports the decision-making process, assessing the consumption of resources and the achievement of expected clinical health outcomes.

## CONCLUSIONS

The total MDC for the joint puncture procedure was US$30.40 (SD=26.36), for nerve block US$14.47 (SD=2.74), for cervical and lumbar spine injections, US$24.92 (SD=23.62), for trigger point inactivation, US$12.90 (SD=2.81), and for interfascial plane block, US$15.88 (SD=11.73).

In the five procedures, there was no significant variation in the MDC with the performers’ DLAB (mean of US$3.93). The largest contribution to the cost composition came from the MDC associated with the consumption of medications, solutions, and materials at US$26.22, US$10.62, US$20.74, US$9.31, and US$12.02, respectively.

The identification of the MDC of the aforementioned procedures contributes, with greater precision, to the assessment of the relationship between the costs and benefits associated with them, offering financial subsidies as one of the aspects to be considered in clinical and administrative decisions aimed at pain treatment/management.

## Data Availability

The research data are available within the article.
