# Supplementary material for: Direct cost of ultrasound-guided outpatient medical procedures for patients with pain
Source: Rev Bras Enferm. 2026 Jun 22;79:e20250317. doi: 10.1590/0034-7167-2025-0317 (PMC13317007; doi:10.1590/0034-7167-2025-0317)
Supplement: Translation

# Translation: Custo direto de procedimentos médicos ambulatoriais guiados por ultrassom destinados a pacientes com dor

**DOI:** 10.1590/0034-7167-2025-0317pt

**Authors:** Otávio Paino Paim, Roberto Del Valhe Abi Rached, Antônio Fernandes Costa Lima

**Affiliations:** I Universidade Santo Amaro. São Paulo, São Paulo, Brasil; II Universidade de São Paulo. São Paulo, São Paulo, Brasil

**Keywords:** Assistência Ambulatorial, Dor Aguda, Dor Crônica, Terapia por Ultrassom, Custos Diretos de Serviços.

## Abstract

**Objetivos:**

mensurar os custos diretos médios relativos à mão de obra e aos insumos requeridos para realização de procedimentos médicos ambulatoriais, guiados por ultrassom, destinados a pacientes com dor.

**Métodos:**

pesquisa de microcusteio quantitativa, exploratório-descritiva, prospectiva, do tipo estudo de caso único, conduzida em clínica de dor de outubro/2024 a janeiro/2025.

**Resultados:**

foram documentados os recursos humanos e insumos (materiais, medicamentos e soluções), consumidos em 82 procedimentos, obtendo-se os seguintes custos diretos médios totais: punção articular - US$30,40 (DP=26,86); bloqueio de nervos - US$14,47 (DP=2,74); infiltrações na coluna cervical e lombar - US$24,92 (DP=23,62); inativação de ponto gatilho - US$12,90 (DP=2,81); e bloqueio interfacial - US$15,88 (DP=11,73). A maior repercussão nos custos decorreu do consumo de medicações/soluções e materiais.

**Conclusões:**

a mensuração dos custos diretos médios dos procedimentos supracitados contribui para avaliação da relação custo-benefício a eles associados, fornecendo subsídios financeiros para decisões clínico-administrativas visando ao adequado tratamento/manejo da dor.

## INTRODUÇÃO

De acordo com a *Internacional Association for the Study of Pain* (IASP), a dor pode ser descrita como uma experiência sensorial e emocional negativa que está relacionada a danos nos tecidos, sejam eles reais ou potenciais^(1)^.

Revisão sistemática, que analisou 35 estudos que investigavam a prevalência de dor crônica no Brasil, evidenciou variação entre 23,02 e 76,17%, com média nacional de 45,59%, afetando mais o sexo feminino. Verificou-se maior prevalência na região Centro-Oeste (56,25%), porém a região com mais estudos e maior população analisada foi a Sudeste (42,2%). Quanto às classificações de mecanismos da IASP, a dor possivelmente nociceptiva obteve prevalência de 36,70%, a dor neuropática, de 14,5%, e a dor nociplástica, de 12,5%^(2)^.

A dor crônica pode ser agravada por doenças psiquiátricas, pois a sensibilização à dor de pacientes com doenças como a depressão é maior que em um indivíduo isento de condições mentais adversas, sendo o estresse contínuo uma influência na dor^(3)^. A secreção excessiva de hormônios de estresse, como cortisol e catecolaminas, é um fator significativo em casos de lombalgia e fibromialgia^(4)^.

A dor aguda é caracterizada por um início súbito, geralmente associada a um evento ou lesão específica^(5)^. Funciona como uma resposta protetora, ativando nociceptores periféricos e transmitindo sinais de dor para o sistema nervoso central^(6)^. Embora geralmente se resolva em um curto período à medida que o corpo se recupera, a dor aguda pode ser severa e dificultar a recuperação. Em alguns casos, se não for manejada de maneira eficaz, pode se transformar em dor crônica, acrescentando desafios para o cuidado e a recuperação do paciente, especialmente em casos pós-cirúrgicos^(7)^.

A complexidade da percepção da dor e a sua natureza multidimensional mostram a importância de uma abordagem abrangente para o seu manejo^(8)^ farmacológico ou por intermédio de procedimentos minimamente invasivos, os quais, a depender do tipo e localização da dor, são considerados mais efetivos^(9)^.

Entre os tratamentos invasivos, a estimulação de nervos periféricos, que tem o potencial de atingir a área acometida, causa diminuição da dor pela estimulação do nervo do tecido lesionado^(10,11)^. Outras técnicas terapêuticas são também eficazes, como bloqueio de plano facetário, injeção em sítios regionais, injeção intra-articular de fármacos e utilização de fármacos anti-inflamatórios com ou sem adição de anestésicos opioides^(11)^.

A fluoroscopia, que utiliza raios-X em tempo real, tornou-se uma ferramenta importante em procedimentos intervencionistas para dor, como bloqueios nervosos e injeções epidurais, permitindo uma visualização precisa e imediata, assim como o ultrassom. Comparando o uso da fluoroscopia e do ultrassom no pós-operatório, a fluoroscopia se mostrou superior no Índice de Incapacidade de Oswestry, que mede as restrições funcionais causadas pela dor em pessoas com lombalgia. Entretanto, tal superioridade não possui implicação clínica significativa, sendo ambos os métodos seguros para reações vasovagais, cefaleia transitória e rubor facial^(12)^.

A radiologia intervencionista desempenha um papel crucial no tratamento de metástases ósseas dolorosas, utilizando técnicas guiadas por ressonância magnética, preservando os tecidos saudáveis, com um tempo de recuperação mais rápido e menos complicações pós-operatórias^(13)^. Revisão sistemática demonstrou que estudos recentes evidenciam a efetividade clínica dos procedimentos guiados por ultrassom no tratamento da dor crônica, tornando os serviços de manejo da dor mais custo-efetivos^(14)^.

Com relação à dor pós-cirúrgica, seu manejo tem sido adaptado de maneira mais precisa às necessidades individuais dos pacientes, por meio da combinação de diferentes métodos terapêuticos, visando reduzir os efeitos colaterais e aumentar o contato rotineiro com os pacientes. Isso promove um tratamento mais eficaz e seguro, com monitoramento e ajuste constantes conforme necessário^(15)^.

As diferentes intervenções para tratar a dor demandam o consumo de mão de obra (MOB) especializada e de insumos (medicamentos, soluções e materiais) que precisam ter seus custos adequadamente apurados e gerenciados, a fim de manter a sustentabilidade financeira da relação estabelecida entre os prestadores de serviços de saúde e as fontes pagadoras, quer sejam do Sistema de Saúde Suplementar (SSS) (seguradoras, operadoras de planos de saúde (OPSs) e/ou pacientes particulares) ou do Sistema Único de Saúde (SUS).

Diante da conjuntura de recursos escassos e demandas crescentes por serviços de saúde, o custeio de processos e/ou procedimentos é fundamental para a eficácia, eficiência e melhoria contínua da qualidade dos sistemas de saúde^(16)^. Auxilia no controle dos custos operacionais, identificando áreas onde há excesso de gastos ou ineficiências, além de permitir a implementação de medidas corretivas visando evitar desperdícios, perdas e obsolescência que resultam em custos desnecessários. Contribui para o processo decisório relativo aos investimentos em novas tecnologias, métodos de tratamento e modelos de atenção mais eficazes e econômicos, desenvolvimento de novos serviços, e incremento de processos^(17,18)^. Nesta perspectiva, realizou-se o presente estudo.

## OBJETIVOS

Mensurar os custos diretos médios (CDMs) relativos à MOB e aos insumos requeridos para realização de procedimentos médicos ambulatoriais, guiados por ultrassom, destinados a pacientes com dor.

## MÉTODOS

### Aspectos éticos

O estudo foi aprovado pelo Comitê de Ética em Pesquisa da Escola de Enfermagem da Universidade de São Paulo.

### Desenho, local do estudo e período da coleta de dados

Pesquisa de microcusteio^(16,19)^ quantitativa, exploratório-descritiva, prospectiva, na modalidade de estudo de caso único^(20)^, conduzida, conforme as diretrizes do instrumento CARE da *Equator Network*, em uma clínica de dor particular localizada no bairro da Bela Vista, cidade de São Paulo.

A clínica de dor campo de estudo foi selecionada por conveniência, tendo em vista possuir adequada infraestrutura para atendimento ambulatorial de pacientes com dor e que um dos autores nela atua como estagiário de medicina. Dispõe de uma sala de procedimentos equipada com tecnologia de imagem avançada para guiar procedimentos intervencionistas, além de consultório para consultas e avaliações médicas.

Atende cerca de 300 pacientes/mês, particulares e provenientes de OPS. Em torno de 80% dos pacientes recebem tratamento com procedimentos guiados por ultrassom. As patologias mais prevalentes são dor cervical e lombar, artrose de joelho, dor miofascial, neuropatias, tendinites, e bursites. Conta com três médicos especialistas em dor e, quando necessário, recomendam ao paciente e seus familiares/responsáveis legais outros profissionais da área de reabilitação (fisioterapeutas, terapeutas ocupacionais, psicólogos, reumatologistas, ortopedistas e psiquiatras) para apoio no tratamento requerido.

Esclarece-se que, entre os médicos, apenas um deles realiza os procedimentos ambulatoriais guiados por ultrassom auxiliados pelo estagiário de medicina. São implementados planos de tratamento individualizados, adaptados às necessidades específicas de cada paciente, com integração de diferentes modalidades terapêuticas visando ao manejo holístico da dor. Além disso, é realizado acompanhamento contínuo com reavaliação dos planos de tratamento para garantir a sua eficácia.

Na clínica de dor, o processo de aquisição de insumos segue um fluxo padronizado visando assegurar a disponibilidade contínua dos materiais necessários e racionalização de custos. A previsão de consumo é elaborada pelo diretor médico, considerando a padronização das quantidades utilizadas em cada procedimento e o histórico de demanda, inclusive para períodos de maior fluxo de atendimentos.

Semanalmente, o estagiário realiza o inventário físico dos insumos remanescentes e encaminha o levantamento à secretária, que formaliza a solicitação de compra por meio de plataformas de *e-commerce* de distribuidores de materiais médicos e de sites de fabricantes específicos para insumos.

Antes da efetivação do pedido, o diretor médico revisa a solicitação para validar as quantidades e aprovar a aquisição. Esse método garante controle rigoroso de estoque, minimiza desperdícios e possibilita negociações mais vantajosas com fornecedores, contribuindo para a sustentabilidade financeira da clínica e manutenção da qualidade assistencial.

No período de coleta de dados, o quantitativo de procedimentos guiados por ultrassom variou de 125 a 189, com média de 151. A coleta de dados ocorreu de outubro de 2024 a janeiro de 2025, e as datas e horários para condução das observações não participantes foram previamente acordados com o médico especialista em dor, respeitando-se os preceitos éticos quanto à intimidade dos pacientes em atendimento e a manutenção do anonimato de todos os envolvidos.

### Amostra, critérios de inclusão e exclusão

A casuística correspondeu às oportunidades de observação direta, não participante, do tempo despendido pelo médico, auxiliado pelo estagiário de medicina, e do consumo de insumos requeridos à viabilização dos seguintes procedimentos médicos ambulatoriais guiados por ultrassom destinados a pacientes com dor: punção articular; bloqueio de nervos; infiltrações na coluna cervical e lombar; inativação de ponto gatilho; e bloqueios interfaciais.

O tamanho amostral de observações, para subsidiar o custeio direto, conforme orientação prévia de profissional estatístico, ocorreu por amostragem não probabilística por conveniência, tendo em vista que os procedimentos seriam executados pelo mesmo médico auxiliado pelo mesmo estagiário; portanto, não haveria variações significativas no tempo despendido pelos executantes (mão de obra direta (MOD)), consumo dos medicamentos, soluções e materiais. Foram incluídos no estudo todos os pacientes tratados no período de coleta de dados.

### Percurso teórico-metodológico

O estudo de caso único foi adotado por subsidiar a investigação empírica de um determinado fenômeno segundo um conjunto de procedimentos. Parte da lógica do planejamento incorpora abordagens específicas referentes às técnicas de coleta e análise de dados advindos de múltiplas fontes de evidências, a fim de compreender os fenômenos contemporâneos a partir do seu contexto real. Propicia apreender a totalidade de uma situação, descrever, compreender e interpretar a complexidade de um caso concreto, aprofundando-se em um objeto delimitado^(20)^.

Fundamentou-se na metodologia de microcusteio, que emprega dados detalhados de utilização de recursos e custos unitários para gerar estimativas precisas de custos econômicos^(16,19)^. Consistiu na aferição dos custos diretos relativos ao consumo de recursos humanos (MOD), materiais, medicamentos e soluções para realização dos procedimentos ambulatoriais guiados por ultrassom.

Definiram-se custos diretos como aqueles que podem ser claramente quantificados e identificados. Dizem respeito a um dispêndio monetário consumido na produção de um produto/serviço no qual é possível a identificação com o produto ou departamento^(21)^. Nas organizações hospitalares, os custos diretos são compostos pela MOD, insumos e equipamentos utilizados no processo assistencial^(22)^.

A execução de um dado procedimento exige o consumo de variadas quantidades de recursos. Calcula-se o CDM de um procedimento a partir da soma do CDM dos materiais, das soluções/medicamentos e da MOB^(23)^.

Para obtenção do CDM total dos procedimentos objetos de estudo, definiram-se a quantidade média dos materiais, o preço unitário médio de cada material,a quantidade média das soluções/medicamentos, o preço unitário médio de cada solução/medicamento, o tempo médio de dedicação de cada categoria profissional e a massa salarial unitária média da MOB de cada categoria profissional^(20)^, conforme a seguinte equação^(23)^: .

Os valores obtidos em reais (R$) foram convertidos para o dólar americano (US$), considerando a taxa média de conversão R$ 5,96/US$ 1.00, relativa ao período de outubro de 2024 a janeiro de 2025.

O diretor médico da clínica de dor disponibilizou as informações relativas aos tipos/apresentações de materiais, e soluções/medicamentos e custos das últimas aquisições, bem como as massas salariais e cargas horárias mensais cumpridas pelo médico (R$20.000,00/150 horas/mês) e estagiário (R$500,00/50 horas/mês), participantes dos procedimentos. Então, obtiveram os valores por hora (R$133,33 e R$10,00) e minuto (R$2,22 e R$0,17).

Após organizados em planilha eletrônica, os dados foram transportados para o simulador de microcusteio direto *bottom-up* ^(24)^, do Grupo de Pesquisa Dimensão Econômica de Gerenciamento em Enfermagem, e tratados por meio de estatística descritiva e inferencial.

## RESULTADOS

Foram realizadas observações não participantes de cinco procedimentos médicos ambulatoriais, guiados por ultrassom, visando ao tratamento de 82 pacientes com dor. As idades desses pacientes variaram de 33 a 85 anos, com média de 58,04 (DP= 15,85) anos. Além disso, 58,03% eram do sexo feminino, e 41,97%, do sexo masculino.

O tempo de duração dos procedimentos variou entre cinco e 13 minutos, com média de 9,62 (DP=1,69) minutos. Houve grande diversidade de diagnósticos médicos, e os mais frequentes, segundo a Classificação Internacional de Doenças-10, corresponderam a: 12 pacientes com G64.1 (outros transtornos do sistema nervoso periférico); 12 pacientes com M53.1 (síndrome cervicobraquial); seis pacientes com M707 (outras bursites do quadril); e cinco pacientes com M54. 5 (dor lombar baixa).

Conforme indica a Tabela 1, o CDM total da punção articular correspondeu a US$30,40 (DP=26,86), variando de US$5,47 a US$77,45. O CDM com a MOD totalizou US$4,18, com destaque para o CDM dos medicamentos, soluções e materiais (US$26,22) representando 86,47% da composição do CDM total.

**Tabela 1:** Distribuição dos custos, em dólares americanos (US$^*^), da mão de obra direta do médico e estagiário de medicina, e dos medicamentos, soluções e materiais consumidos em 17 procedimentos de punção articular, guiados por ultrassom, destinados a pacientes com dor, São Paulo, São Paulo, Brasil, 2025

Variáveis	Média US$^*^	Desvio padrão US$^*^	Mínimo US$^*^	Máximo US$^*^	Mediana US$^*^
Custo com mão de obra direta do médico	3,82	0,55	2,61	4,85	3,73
Custo com mão de obra direta do estagiário	0,36	0,05	0,24	0,45	0,35
Custo direto médio com medicamentos, soluções e materiais	26,22	26,81	2,61	73,37	10,13
**Custo direto médio total**	**30,40**	**26,86**	**5,47**	**77,45**	**14,70**

* *Banco Central do Brasil: taxa média de conversão de R$5,96/US$1,00 relativa a outubro de 2024 a janeiro de 2025.*

Nas 17 punções articulares observadas, prevaleceram os custos associados ao consumo dos medicamentos *Osteonil Plus* ^®^ injetável 40 mg (valor unitário de US$49,33), *Synollis* ^®^ 40/80 mg/ml (US$65,44), Triancil^®^ 20 mg/mL (valor unitário de US$4,66), e cloridrato de levobupivacaína em excesso de enantiomérico sem vasoconstritor 0,50% 5 mg/mL (valor unitário de US$4,53).

Na Tabela 2, verifica-se que o CDM total do bloqueio de nervos correspondeu a US$14,47 (DP=2,74), com mínimo de US$11,58 e máximo de US$21,03. O CDM com a MOD foi de US$3,86, destacando-se o CDM dos medicamentos, soluções e materiais (US$10,62), que corresponderam a 73,73% da composição do CDM total.

**Tabela 2:** Distribuição dos custos, em dólares americanos (US$^*^), da mão de obra direta do médico e estagiário de medicina, e dos medicamentos, soluções e materiais consumidos em 16 procedimentos de bloqueio de nervos, guiados por ultrassom, destinados a pacientes com dor, São Paulo, São Paulo, Brasil, 2025

Variáveis	Média US$^*^	Desvio padrão US$^*^	Mínimo US$^*^	Máximo US$^*^	Mediana US$^*^
Custo com mão de obra direta do médico	3,50	0,53	2,61	4,47	3,54
Custo com mão de obra direta do estagiário	0,36	0,05	0,24	0,45	0,35
Custo direto médio com medicamentos, soluções e materiais	10,62	2,60	7,52	16,99	9,62
**Custo direto médio total**	**14,47**	**2,74**	**11,58**	**21,03**	**13,57**

* *Banco Central do Brasil: taxa média de conversão de R$5,96/US$1,00 relativa a outubro de 2024 a janeiro de 2025.*

Nos 16 bloqueios de nervos observados, predominaram os custos com os medicamentos cloridrato de levobupivacaína em excesso de enantiomérico sem vasoconstritor 0,50% 5 mg/mL - 20 mL (valor unitário de US$4,53), cloridrato de lidocaína sem vasoconstritor 2,0% 20 mg/mL (valor unitário de US$1,69) e glicose 50% - 10 ml (valor unitário de US$0,80). Entre os materiais, os maiores custos decorreram do consumo de agulhas 25G x 60 *Nanoneedle Japanese Steel* ^®^ (valor unitário de US$2,25).

De acordo com a Tabela 3, o CDM total correspondeu a US$24,92 (DP=23,62), variando entre US$6,78 e US$85,59. O CDM com a MOD totalizou US$4,18, sendo o CDM dos medicamentos, soluções e materiais (US$20,74 - 83,46%) o mais representativo na composição do CDM total.

**Tabela 3:** Distribuição dos custos, em dólares americanos (US$^*^), da mão de obra direta do médico e estagiário de medicina, e dos medicamentos, soluções e materiais consumidos em 16 procedimentos de infiltrações na coluna cervical e lombar, guiados por ultrassom, destinados a pacientes com dor, São Paulo, São Paulo, Brasil, 2025

Variáveis	Média US$^*^	Desvio padrão US$^*^	Mínimo US$^*^	Máximo US$^*^	Mediana US$^*^
Custo com mão de obra direta do médico	3,82	0,60	2,98	4,85	3,73
Custo com mão de obra direta do estagiário	0,36	0,06	0,28	0,45	0,35
Custo direto médio com medicamentos, soluções e materiais	20,74	23,64	2,70	81,92	10,87
**Custo direto médio total**	**24,92**	**23,62**	**6,78**	**85,59**	**13,56**

* *Banco Central do Brasil: taxa média de conversão de R$5,96/US$1,00 relativa a outubro de 2024 a janeiro de 2025.*

Nas 16 infiltrações na coluna cervical e lombar observadas, sobressaiu-se o custo referente ao consumo dos medicamentos Triancil^®^ 20 mg/mL (valor unitário de US$4,66), cloridrato de levobupivacaína em excesso de enantiomérico sem vasoconstritor 0,50% 5 mg/mL (valor unitário de US$4,53) e acetato de metilprednisolona 40 mg/mL - 2 mL (valor unitário de US$4,51).

Segundo a Tabela 4, o CDM total correspondeu a US$12,90 (DP= US$2,81), com mínimo de US$7,06 e máximo de US$18,03. O CDM com a MOD totalizou US$3,59, com predomínio do CDM dos medicamentos, soluções e materiais (US$9,31) representando 72,48% da composição do CDM total.

**Tabela 4:** Distribuição dos custos, em dólares americanos (US$^*^), da mão de obra direta do médico e estagiário de medicina, e dos medicamentos, soluções e materiais consumidos em 16 procedimentos de inativação de ponto gatilho, guiados por ultrassom, destinados a pacientes com dor, São Paulo, São Paulo, Brasil, 2025

Variáveis	Média US$^*^	Desvio padrão US$^*^	Mínimo US$^*^	Máximo US$^*^	Mediana US$^*^
Custo com mão de obra direta do médico	3,28	0,80	1,86	4,47	3,54
Custo com mão de obra direta do estagiário	0,31	0,07	0,17	0,42	0,33
Custo direto médio com medicamentos, soluções e materiais	9,31	2,90	2,98	13,95	8,83
**Custo direto médio total**	**12,90**	**2,81**	**7,06**	**18,03**	**12,60**

* *Banco Central do Brasil: taxa média de conversão de R$5,96/US$1,00 relativa a outubro de 2024 a janeiro de 2025.*

Nas 16 inativações de pontos gatilho observadas, prevaleceram os custos com os medicamentos cloridrato de levobupivacaína em excesso de enantiomérico sem vasoconstritor 0,50% 5 mg/mL (valor unitário de US$4,53) e cloridrato de lidocaína sem vasoconstritor 2,0% 20 mg/mL (valor unitário de US$1,69). Quanto aos materiais, os maiores custos se associaram ao consumo de agulhas 25G x 60 *Nanoneedle Japanese Steel* ^®^ (valor unitário de US$2,25) e de anestesia Raquidiana Spinal Bd^®^ - 25G x 3 1/2 (valor unitário de US$1,64).

Por fim, consta da Tabela 5 que o CDM total do bloqueio interfacial foi de US$15,88 (DP= US$11,73), variando de US$6,02 a US$59,54. O CDM com a MOD correspondeu a US$3,86, e o CDM dos medicamentos, soluções e materiais (US$12,02) representou 76% da composição do CDM total.

**Tabela 5:** Distribuição dos custos, em dólares americanos (US$^*^), da mão de obra direta do médico e estagiário de medicina, e dos medicamentos, soluções e materiais consumidos em 17 procedimentos de bloqueio interfacial, guiados por ultrassom, destinados a pacientes com dor, São Paulo, São Paulo, Brasil, 2025

Variáveis	Média US$^*^	Desvio padrão US$^*^	Mínimo US$^*^	Máximo US$^*^	Mediana US$^*^
Custo com mão de obra direta do médico	3,53	0,53	2,61	4,47	3,73
Custo com mão de obra direta do estagiário	0,33	0,05	0,24	0,42	0,35
Custo direto médio com medicamentos, soluções e materiais	12,02	11,54	2,35	55,05	9,84
**Custo direto médio total**	**15,88**	**11,73**	**6,02**	**59,54**	**13,24**

* *Banco Central do Brasil: taxa média de conversão de R$5,96/US$1,00 relativa a outubro de 2024 a janeiro de 2025.*

Nos 17 bloqueios interfaciais observados, predominaram os custos com os medicamentos cloridrato de levobupivacaína em excesso de enantiomérico sem vasoconstritor 0,50% 5 mg/mL - 20 mL (valor unitário de US$4,53), *Osteonil Plus* ^®^ injetável 40 mg (valor unitário de US$49,33), cloridrato de lidocaína sem vasoconstritor 2,0% 20 mg/mL (valor unitário de US$1,69), e com o material agulhas 25G x 60 *Nanoneedle Japanese Steel* ^®^ (valor unitário de US$2,25).

## DISCUSSÃO

A condução deste estudo de caso único propiciou analisar os procedimentos médicos ambulatoriais, guiados por ultrassom, destinados a pacientes atendidos em uma clínica de dor. Conforme previsto nesta abordagem^(20)^, obtiveram-se informações pertinentes, considerando a especificidade do seu contexto real para direcionar a tomada de decisão clínica, averiguar a necessidade do desenvolvimento de novas estratégias de cuidado e contribuir com a melhoria da prática profissional.

Os pacientes integrantes do estudo apresentaram média de idade de 58,04 (DP=15,85) anos, sendo 58,03% do sexo feminino, com diagnósticos clínicos variados, predominando os transtornos musculoesqueléticos e neurológicos periféricos. Destacaram-se as classificações G64.1 (outros transtornos do sistema nervoso periférico), M53.1 (síndrome cervicobraquial), M70.7 (outras bursites do quadril) e M54.5 (dor lombar baixa). Tais diagnósticos correspondem a quadros clássicos de dor musculoesquelética e neuropática, frequentemente observados em contextos de reabilitação.

O perfil acima mencionado vai ao encontro dos resultados de revisão sistemática, realizada no Brasil, que evidenciou maior prevalência de dor crônica entre mulheres, especialmente com idades entre 45 e 66 anos^(2)^. Outra revisão sistemática e metanálise verificou a prevalência de dor crônica na população adulta em geral, entre 23,02% e 41,4%, associada ao sexo feminino, com idade avançada (de 29,3% a 76,2%). Concluiu que a dor crônica é altamente prevalente, e está associada ao sofrimento significativo e incapacidade, e tem sido mal controlada^(25)^.

A média etária da amostra analisada reflete uma fase da vida em que se intensificam alterações degenerativas musculoesqueléticas e neurológicas periféricas, frequentemente associadas à limitação funcional. Esses achados ressaltam a necessidade de abordagens terapêuticas específicas direcionadas ao manejo da dor nesse perfil etário^(26)^. Estudo enfatiza que condições como tendinopatias, bursites e lombalgias são altamente prevalentes, e tendem a apresentar boa resposta a terapias locais^(11)^. Mulheres com fibromialgia, mesmo aquelas com maior nível de escolaridade, enfrentam impactos funcionais e emocionais significativos decorrentes da dor crônica^(27)^, reforçando a pertinência de terapias específicas, como os bloqueios e infiltrações custeados neste estudo.

Na apuração dos CDMs totais, não houve variação significativa do CDM com MOD do médico especialista em dor e do estagiário graduando em medicina, a qual variou de R$3,59 a US$4,18, com média de US$3,93. Apesar da imprescindibilidade da MOD especializada dos executantes, este CDM correspondeu à menor representatividade na composição do CDM total dos procedimentos objetos de estudo, corroborando resultados de estudos de custeio direto que demonstraram a prevalência dos custos com materiais e/ou medicamentos/soluções^(17,18)^.

Destacaram-se os custos da punção articular (US$30,40) e os da inativação de pontos gatilho (US$12,90), os quais apresentaram o maior e menor CDM total. Justifica-se essa disparidade devido à complexidade do atendimento e à necessidade de materiais específicos que impactam, diretamente, o custo das intervenções ambulatoriais^(28)^. No caso das punções articulares, o maior custo se relacionou ao uso de medicamentos específicos como o *Osteonil Plus* ^®^. A dependência de fármacos de alto custo reforça a necessidade de políticas públicas que favoreçam a aquisição desses insumos com preços acessíveis. Com essa intencionalidade, a gestão eficiente de compras hospitalares pode reduzir, significativamente, o custo unitário de materiais de alto valor agregado^(29)^.

Quanto aos bloqueios de nervos, apesar de também requererem medicamentos como a levobupivacaína e a lidocaína, o CDM foi relativamente baixo (US$14,47), indicando um bom custo-benefício deste procedimento e considerando, inclusive, sua eficácia terapêutica relatada em literatura. Revisão integrativa evidencia que procedimentos guiados por ultrassonografia proporcionam alívio significativo da dor, reduzem a necessidade de opioides e melhoram a recuperação funcional dos pacientes^(14)^.

As infiltrações na coluna obtiveram um CDM elevado (US$24,92), com variação entre os casos observados (DP=US$23,62), o que aponta à heterogeneidade na escolha terapêutica ou na resposta individual ao tratamento. Isso se alinha ao observado em estudo que destaca a importância da personalização terapêutica em tratamentos intervencionistas da dor, ainda que isso represente o enfrentamento de desafios no controle dos custos^(30)^.

A inativação de pontos gatilho e os bloqueios interfaciais se destacaram por apresentar custos mais uniformes e, relativamente, mais baixos. No entanto, reitera-se que, nos cinco procedimentos estudados, os CDMs com medicamentos, soluções e materiais predominaram na composição dos CDMs totais.

Então, corrobora-se a relevância do adequado gerenciamento dos recursos materiais para identificar, analisar e reduzir os desperdícios e perdas resultantes em custos que, caso evitados, poderiam ser revertidos para o benefício de outros pacientes, contribuindo para a eficiência dos processos e sustentabilidade financeira das organizações de saúde^(31)^. Nessa direção, estratégias de racionalização na aquisição e prescrição de medicamentos são imprescindíveis^(32)^.

Sabe-se que procedimentos mais complexos, que requerem MOD especializada e maior quantidade de materiais, medicamentos e soluções, resultam em custos mais elevados. Portanto, é necessário considerar a complexidade dos procedimentos e os recursos específicos no planejamento e gerenciamento de intervenções ambulatoriais, visando à sua otimização e contribuição com a sustentabilidade financeira dos serviços de saúde. Assim, o planejamento cuidadoso da logística dos recursos requeridos, aliado à sua alocação e uso racional, pode favorecer a ampliação do acesso a essas terapias no âmbito ambulatorial. Tal perspectiva é coerente com os princípios da economia da saúde, que visam à otimização dos recursos com base na efetividade clínica^(28)^.

Revisão sistemática evidenciou que estudos recentes têm ressaltado a efetividade clínica dos procedimentos guiados por ultrassom no tratamento da dor crônica. A literatura indica que os serviços de manejo da dor podem ser custo-efetivos para tratamento da dor lombar crônica, ainda que haja variabilidade nas evidências devido à qualidade dos estudos publicados e à diversidade de intervenções, comparadores e desfechos^(33)^.

O custo dos fármacos utilizados, especialmente os de alto custo (*Osteonil Plus* ^®^ e o *Synollis* ^®^), influenciou, significativamente, os CDMs das punções articulares neste estudo. Destaca-se que esquemas de compartilhamento de custos, preços baseados em valor, preços de referência, mecanismos de reembolso e a substituição de medicamentos originais por genéricos e biossimilares têm se constituído em estratégias para gerenciar os gastos com produtos farmacêuticos, garantindo acesso justo a medicamentos essenciais^(34)^.

No SSS brasileiro, após avaliação de 79.689 pacientes com dor crônica ao longo de quatro anos, identificou-se que 96% dos pacientes realizaram pelo menos um exame, e 86% tiveram pelo menos uma consulta de emergência durante o período. O custo total estimado para o SSS correspondeu a mais de 3 bilhões de reais. Especialistas apontaram que a fragmentação do sistema de saúde e a falta de abordagens centradas no paciente contribuem para o manejo ineficaz da dor, resultando em alto uso de recursos. Enfatizaram a importância da integração de dados clínicos e de custos diretos para gestão mais eficiente dos recursos e ampliação do acesso a terapias intervencionistas^(32)^.

Revisão temática da literatura demonstrou que as implicações gerenciais da precificação de serviços de saúde, especialmente hospitais, ainda têm recebido atenção acadêmica insuficiente. Ao analisar a compreensão da natureza da precificação de serviços hospitalares, a diferenciação de objetivos, estratégias e práticas de precificação, e a apresentação dos fatores que impactam a precificação de serviços hospitalares, indicou-se que a precificação hospitalar é uma questão complexa e pouco clara. Tem sido influenciada por custos, fatores de demanda e oferta, estrutura de mercado, regulamentação de preços e reembolsos de terceiros^(35)^.

Por fim, corrobora-se que o método de microcusteio, frequentemente considerado “padrão-ouro” devido à sua capacidade de fornecer dados de custos detalhados e precisos, permite a análise minuciosa de cada componente individual de custo de um serviço ou tratamento, abrangendo medicamentos, materiais, equipamentos e MOB, resultando em informações mais confiáveis, e possibilita uma avaliação com alto nível de precisão em relação ao processo ou procedimento. Essa precisão é crucial para avaliação econômica de tecnologias em saúde, permitindo identificar quais tratamentos ou intervenções são mais custo-efetivos^(16)^.

### Limitações do estudo

A realização de um estudo de caso único consistiu na limitação deste estudo. Para investigações futuras, e verticalização do conhecimento desenvolvido, recomendam-se estudos de casos múltiplos, os quais possibilitarão a abrangência de múltiplas dimensões e perspectivas, cuja comparação fortalecerá a robustez dos resultados.

### Contribuições para as áreas da enfermagem, saúde ou políticas públicas

Os resultados obtidos contribuem para proposição de uma base racional para auxiliar aos administradores de serviços de saúde a fundamentar o processo de precificação de procedimentos médicos ambulatoriais, guiados por ultrassom, destinados a pacientes com dor. Nesta perspectiva, favorece a negociação junto às fontes pagadoras, sejam elas do SSS, SUS ou pacientes particulares.

A metodologia adotada no presente estudo poderá ser replicada em outros serviços de saúde, independentemente da sua natureza jurídica, bem como para procedimentos executados por outras categorias profissionais de saúde, fornecendo informações financeiras que fundamentem o processo decisório, avaliando o consumo de insumos e o alcance dos desfechos clínicos em saúde esperados.

## CONCLUSÕES

Os CDMs totais do procedimento punção articular corresponderam a US$30,40 (DP=26,36), do bloqueio de nervos, a US$14,47 (DP=2,74), das infiltrações na coluna cervical e lombar, a US$24,92 (DP=23,62), da inativação de ponto gatilho, a US$12,90 (DP=2,81), e do bloqueio interfacial, a US$15,88 (DP=11,73).

Nos cinco procedimentos, não houve variação significativa do CDM com a MOD dos executantes (média de US$3,93). A maior participação na composição dos custos decorreu do CDM associado ao consumo de medicações, soluções e materiais a US$26,22, US$10,62, US$20,74, US$9,31 e US$12,02, respectivamente.

A identificação dos CDMs dos procedimentos supracitados contribui, com maior precisão, para avaliação da relação entre os custos e os benefícios a eles associados, oferecendo subsídios financeiros como um dos aspectos a serem considerados nas decisões clínicas e administrativas visando ao tratamento/manejo da dor.

## Data Availability

Os dados de pesquisa estão disponíveis no corpo do artigo.
